# Effect of COVID-19 pandemic on provision of sexual and reproductive health services in primary health facilities in Nigeria: a cross-sectional study

**DOI:** 10.1186/s12978-021-01217-5

**Published:** 2021-08-04

**Authors:** Babatunde Adelekan, Erika Goldson, Zubaida Abubakar, Ulla Mueller, Audu Alayande, Tellson Ojogun, Lorretta Ntoimo, Bukky Williams, Ibrahim Muhammed, Friday Okonofua

**Affiliations:** 1 United Nations Population Fund (UNFPA) Country Office, Abuja, Nigeria; 2 Women’s Health and Action Research Centre (WHARC), Km 11 Benin-Lagos Expressway, Igue-Iheya, Benin City, Edo Nigeria; 3 Department of Demography and Social Statistics, Federal University Oye-Ekiti, Oye, Nigeria; 4 Centre of Excellence in Reproductive Health Innovation (CERHI), University of Benin, Benin City, Nigeria; 5 Department of Obstetrics and Gynaecology, School of Medicine, University of Benin, Benin City, Nigeria; 6 Education as a Vaccine (EVA), Abuja, Nigeria; 7 Planned Parenthood Federation of Nigeria (PPFN), Abuja, Nigeria

**Keywords:** COVID-19, Pandemic, Primary Health Facilities, Reproductive Health, Maternal and Child Health, Adolescent Health, Nigeria

## Abstract

**Background:**

Nigeria, like many other countries, has been severely affected by the COVID-19 pandemic. While efforts have been devoted to curtailing the disease, a major concern has been its potential effects on the delivery and utilization of reproductive health care services in the country. The objective of the study was to investigate the extent to which the COVID-19 pandemic and related lockdowns had affected the provision of essential reproductive, maternal, child, and adolescent health (RMCAH) services in primary health care facilities across the Nigerian States.

**Methods:**

This was a cross-sectional study of 307 primary health centres (PHCs) in 30 Local Government Areas in 10 States, representing the six geopolitical regions of the country. A semi-structured interviewer-administered questionnaire was used to obtain data on issues relating to access and provision of RMCAH services before, during and after COVID-19 lockdowns from the head nurses/midwives in the facilities. The questionnaire was entered into Open Data Kit mounted on smartphones. Data were analysed using frequency and percentage, summary statistics, and Kruskal–Wallis test.

**Results:**

Between 76 and 97% of the PHCS offered RMCAH services before the lockdown. Except in antenatal, delivery and adolescent care, there was a decline of between 2 and 6% in all the services during the lockdown and up to 10% decline after the lockdown with variation across and within States. During the lockdown. Full-service delivery was reported by 75.2% whereas 24.8% delivered partial services. There was a significant reduction in clients’ utilization of the services during the lockdown, and the difference between States before the pandemic, during, and after the lockdown. Reported difficulties during the lockdown included stock-out of drugs (25.7%), stock-out of contraceptives (25.1%), harassment by the law enforcement agents (76.9%), and transportation difficulties (55.8%). Only 2% of the PHCs reported the availability of gowns, 18% had gloves, 90.1% had hand sanitizers, and a temperature checker was available in 94.1%. Slightly above 10% identified clients with symptoms of COVID-19.

**Conclusions:**

The large proportion of PHCs who provided RMCAH services despite the lockdown demonstrates resilience. Considering the several difficulties reported, and the limited provision of primary protective equipment more effort by the government and non-governmental agencies is recommended to strengthen delivery of sexual and reproductive health in primary health centres in Nigeria during the pandemic.

**Supplementary Information:**

The online version contains supplementary material available at 10.1186/s12978-021-01217-5.

## Plain language summary

The onset of COVID-19 has raised concerns that it may compromise women’s access to sexual and reproductive health and rights. Although data are still emerging, some reports indicate reduced access to sexual and reproductive health services, largely due to disruptions in the demand and supply of contraceptive commodities, the diversion of staff and resources to other clinical services, and clinic closures. While these concerns have similarly been broached for Nigeria, there has been no systematic documentation of the extent of the disruptions of reproductive health services caused by COVID-19 and its effects on the provision and utilization of related services in the country This study was a cross-sectional facility-based survey conducted in 10 states, 30 Local Government Areas and 302 primary health centres in Nigeria. The objective of the study was to explore through key informant interviews with service providers in the health centres, the effects of the COVID-19 pandemic on demand and supply of sexual and reproductive health services. Field assistants administered a semi-structured interview guide to the heads of the health centres that elicited information on availability and use of the health centres before, during and after the lock-downs associated with the pandemic. The results indicate that a large proportion of the health centres opened for the provision of essential sexual and reproductive health services during the COVID-19 pandemic lockdown. However, fewer clients used the services due to difficulties in travel because of the lockdowns, stock-outs in the health centres, and the fear that they may contract the virus if they leave their houses to the health centres. Although the health centres reported some cases of COVID-19, there was limited provision for personal protective equipment to motivate the health workers to optimize services for clients. From this study, we conclude that efforts should be made to identify innovations for addressing these challenges to enable the continued provision of sexual and reproductive health services by health centres despite the COVID-19 pandemic in Nigeria’s health centres.

**Supplementary Information:**

The online version contains supplementary material available at 10.1186/s12978-021-01217-5.

## Introduction

Nigeria, like many other countries, has been severely affected by the COVID-19 pandemic. The first case of the disease was reported in the country in February 2020 [1]. The number of cases further surged in the country in March 2020 coincident with its declaration as a pandemic by the World Health Organization [2]. Arising from this development, the Nigerian government constituted a Presidential Task Force (PTF), which was saddled with the task of identifying all cases of COVID-19, establishing accurate surveillance of contacts, and ensuring the isolation and treatment of those infected [3]. The PTF thereafter swung into action by a proclamation of lockdown of all sectors of the country and established a system of daily reporting of cases and deaths from the disease [4].

Despite these efforts, the progression in the number of cases of COVID-19 in the country has not abated. To date (July 1, 2021), 7.3% (167,618) of 2,300,266 samples tested have been confirmed cases of the virus, with a case fatality rate of 1.3% (2, 120) thus, making Nigeria the 4^th^ most affected country in Africa [5]. The pandemic has no doubt affected several sectors of the Nigerian economy including the educational, health, and agricultural sectors, resulting in a negative overall economic growth of the country in the latter half of 2020 [6]. Most worrisome is its effects on the already precarious health care delivery system in Nigeria. While efforts have been devoted to curtailing the disease, a major concern relating to its effects on potential neglect of other essential services has featured in several reviews and publications [7, 8]. For a country already witnessing dismal performances in essential health indicators, this neglect has been identified as a major challenge for health and social development in the country.

More specifically, COVID-19 has been postulated to pose a challenge to women’s access to sexual and reproductive health and rights (SRHR) [9–11]. Although data are still emerging, a recent survey of frontline health workers in 81 countries reported significant reductions in the use of sexual and reproductive health (SRH) services [12–14], largely due to disruptions in the production and supply of contraceptive commodities [15], diversion of staff and resources to urgent clinical care, clinic closures, and travel restrictions [16]. While these have similarly been projected for Nigeria, there has been no systematic documentation of the extent of the disruptions caused by COVID-19 on the provision and utilization of services, especially those related to reproductive, maternal, child and adolescent health in the country.

Nigeria has some of the most daunting statistics relating to maternal and child health, reproductive health (especially fertility control and gender-based violence). and adolescent reproductive health in the world [17–19]. Nigeria contributed 23% of the global maternal deaths in 2017 with a total of 67,000 deaths, and with India contributed almost a third of under-five deaths in 2019 [17, 18]. Only 12% of women of reproductive age in the country use any modern method of contraception, and 1 in 3 women have ever experienced physical violence [19]. It is important that while COVID-19 is being curtailed that measures are put in place to ensure that essential services that promote all components of reproductive health, maternal and child health, and adolescent reproductive health continue to be implemented in all parts of the country. This would eliminate the possibility that the pandemic could reduce the gains that have been made in promoting all elements of comprehensive reproductive health in the country. Although Nigeria did not achieve the MDG goals on maternal and child health, some gains have been made over time. For instance, there was a 39.7% change in maternal mortality ratio from 1350/100000 live births in 1990 to 814 in 2015 [20]. The percentage of women aged 15–49 who received antenatal care from a skilled provider increased from 57% in 1990 to 67% in 2018, and the proportion of births assisted by skilled birth attendants increased from 39% in 2008 to 43% in 2018 [19]. Also, under-five mortality declined from 210/1000 live births in 1990 to 117 in 2019, indicating a 2.0% annual rate of change [18].

The objective of this study was to investigate the extent to which the COVID-19 pandemic and related lockdowns had affected the provision and utilization of essential reproductive health, maternal and child health, and adolescent health services in primary health facilities, and the challenges in service delivery across ten Nigerian States. We believe the results would be useful in planning the comprehensive delivery of resilient SRHR services in Nigeria in ways to enable them to overcome the fragilities posed by COVID-19.

## Method

### Design and setting

The study was part of a bigger intervention initiated by the UNFPA and implemented by three non-governmental organizations (NGOs): The Women’s Health and Action Research Centre (WHARC), Education as a Vaccine (EVA), and the Planned Parenthood Federation of Nigeria (PPFN). The three implementation partners (IPs) worked in partnership with three identified Civil Society Organizations (CSOs) per State to conduct the study. Overall, 30 CSOs worked with WHARC, EVA and the PPFN to conduct the study. The design was a cross-sectional descriptive study conducted in selected Primary Health Centres (PHCs) in two–three purposefully selected Local Government Areas (LGAs) in 10 States in Nigeria. The states were Lagos, Akwa Ibom, Kano, Kaduna, Gombe, Borno, Ogun, Enugu, Adamawa, and the Federal Capital Territory (FCT) (Abuja Municipal Area Council). The states were drawn from the six geopolitical zones or region of Nigeria (North Central, North East, North West, South East, South-South, and South West). A total of 32 PHCs were purposefully selected from the LGAs in each State, making a total of 320 health facilities. The head nurse/midwife (or Deputy) in each PHC was the respondent. The respondents were all female who had attained national training qualifications and registration requirements with the Nigerian Nursing and Midwifery Council. The exclusion criteria were non-functional, and inaccessible (due to security reasons) PHCs before and after the pandemic started. A total of 307 PHCs were successfully assessed (a non-response rate of 4.1%). The prevalence of COVID-19 cases informed the selection of States and LGAs. States with a relatively higher prevalence of COVID-19 cases were selected. With assistance from the Ministry of Health, and the State Primary Health Care Development Agency in each state, LGAs with high prevalence and the functional and accessible PHCs in the LGAs were identified and selected for the study. We ensured a mix of rural, semi-urban and urban LGAs.

### Data collection

The study protocol was developed in WHARC and revised and finalised by all IPs. Thereafter, each CSO identified the respondents for health facilities in each State. The data collectors were trained by the IPs in the art of collecting quantitative survey data.

The data were collected from November 1 to December 16, 2020, with a questionnaire that was programmed into the Open Data Kit (ODK) for interviewer-administered computer-assisted personal interviewing. The weekly records from each service was sighted and reviewed weekly. The questionnaire contained basic questions on the description of the health facility, maternal, child, adolescent health service delivery and utilization, and difficulties experienced before, during and after the COVID-19 pandemic lockdown. The COVID-19 lockdown took place in Nigeria in mid-March 2020 and was eased in September 2020. Thus, the period before the lockdown was identified as any time before March 15, 2020, while the lockdown period was from March 15 to the end of September 2020. The period after September 2020 when no lock-down occurred, and all schools, markets and Churches were re-opened to users was defined as the post lockdown period.

Specific questions were asked on service delivery before, during and after the lockdowns. The specific services whose functionality were investigated were family planning, antenatal care, delivery (intrapartum) care, immunization services, and adolescent reproductive health services. The details of these services are as provided in the national guidelines for PHC system in Nigeria [21] The respondents were asked what reproductive, maternal, child, and adolescent and adolescent health (RMNCH) services they provided before the pandemic started, during the lockdown, and after the lockdown. The response was a multiple choice 8-item list which included family planning, antenatal, delivery, postnatal, child immunization, childcare, adolescent care, and others (to be specified). Response was also solicited on the closure of the facilities during the lockdown and whether services were offered fully or partially, the number of clients per week (records were sighted), difficulties in service delivery such as stock-outs and transportation, harassment by law enforcement agents (undue delay and questioning by the police or other law enforcement agents), the availability of personal protective equipment, and the identification and management of persons with suspected symptoms of COVID-19.

### Data analysis

The data were extracted from the ODK to SPSS PC + software for data cleaning and analysis. The descriptive results are presented as absolute numbers, percentage, mean and standard deviation, and range where appropriate. Further analysis to determine a statistically significant difference between States in the number of clients utilizing each service before the pandemic, during and after the lockdown was conducted. The distribution for each service was not normal and the Levene test of homogeneity of variances was also violated for each service. Thus, the non-parametric alternative of one-way between-groups analysis of variance (Kruskal–Wallis test) was used to determine whether there was a significant difference by State. The alpha was set at 0.05.

### Ethical approval

The Ministries of Health in the ten States provided permission and ethical approval to undertake the study in the states. Each Ministry was approached differently and informed of the purpose of the study. The research teams then had meetings with responsive officers who reviewed the study protocol in detail and provided ethical approvals through their ethical review committees. The Local Government officers in charge of the PHCs also provided approval, while further consent was obtained from the lead officers in each PHCs. Only officials who accepted to complete the fully explained protocol were finally included in the study. They were assured of confidentiality of information they provide and also that their names would not feature in the protocol or anywhere in the study report.

## Results

The distribution of the PHCs by State is presented in Table 1. The PHCs were selected from 30 LGAs, three per state, with all being rural or semi-urban, and urban. The shortfall was in Borno State where 20 instead of 32 PHCs were accessed, due to the ongoing insurgency which has reduced movements to many health facilities in the state.

**Table 1:** Number of studied PHC facilities by State

Geo-political zone	State	LGA	Freq	Percent
South-South	Akwa Ibom	Ikot Ekpene (semi-urban/rural) Uyo (urban) Eket (rural/semi-urban)	32	10.4
North –East	Borno	Maiduguri (urban/semi-urban) Konduga (rural) Jere (urban/semi-urban/rural)	20	6.5
South East	Enugu	Enugu South (urban/semi-urban) Enugu North (urban/semi-urban) Udi (rural/semi-urban)	32	10.4
North East	Gombe	Gombe (urban/semi-urban) Akko (urban/Semi-urban/Rural)	33	10.7
North Central	Kaduna	Chikun (semi-urban/rural/urban) Kaduna North (urban/semi-urban/rural)	38	12.4
North West	Kano	Nassarawa (urban) Tarauni (urban)	30	9.8
South West	Lagos	Eti-Osa (urban) Alimosho (urban) Ikeja (urban)	32	10.4
South West	Ogun	Abeokuta South (urban/semi-urban) Shagamu (urban) Ado-Odo Ota (urban)	31	10.1
North-west	Sokoto	Dange Shuni (rural) Sokoto South (urban/semi-urban) Wammako (urban/semi-urban/rural)	32	10.4
North Central	FCT	Abuja Municipal Area Council (urban/semi-urban/rural) Bwari (urban/semi-urban/rural)	27	8.8
	Total		307	100.0

### SRHR service delivery before the pandemic, during, and after the pandemic lockdown

The percentage distribution of facilities offering RMNCH services before the pandemic, during, and after the pandemic lockdown is presented in Table 2.

**Table 2:** Percentage distribution of facilities offering RMNCH services before, during, and after the pandemic lockdown

Family planning			
State	Before pandemic (%)	During lockdown (%)	After lockdown (%)
Akwa Ibom	100	100.0	90.6
FCT	100	100.0	96.3
Borno	90.0	90.0	90.0
Enugu	96.9	93.8	81.2
Gombe	90.9	90.9	87.9
Kaduna	97.4	100.0	94.7
Kano	100	96.7	100.0
Lagos	100	93.8	96.9
Ogun	100	90.3	93.5
Sokoto	100	100	93.8
Total	97.7	95.8	92.5
Antenatal care			
Akwa Ibom	100.0	100.0	100.0
FCT	100.0	100.0	100.0
Borno	90.0	90.0	90.0
Enugu	90.6	90.6	96.9
Gombe	97.0	87.9	97.0
Kaduna	97.4	100.0	100.0
Kano	100.0	100.0	100.0
Lagos	90.6	93.8	96.9
Ogun	87.1	87.1	93.5
Sokoto	93.8	96.9	100.0
Total	94.8	94.8	97.7
Delivery care			
Akwa Ibom	90.6	90.6	100.0
FCT	100.0	100.0	100.0
Borno	85.0	90.0	90.0
Enugu	78.1	81.2	87.5
Gombe	93.9	97.0	93.9
Kaduna	94.7	97.4	100.0
Kano	76.7	76.7	100.0
Lagos	37.5	40.6	84.4
Ogun	74.2	77.4	87.1
Sokoto	87.5	87.5	96.9
Total	81.8	83.7	94.1
Postnatal care			
Akwa Ibom	93.8	90.6	90.6
FCT	96.3	92.6	100.0
Borno	90.0	90.0	90.0
Enugu	87.5	81.2	78.1
Gombe	90.9	84.8	90.9
Kaduna	97.4	100.0	97.4
Kano	73.3	70.0	76.7
Lagos	78.1	81.2	40.6
Ogun	80.6	80.6	77.4
Sokoto	100.0	96.9	87.5
Total	88.9	87.0	82.7
Childhood immunization			
Akwa Ibom	100.0	93.8	93.8
FCT	100.0	100.0	96.3
Borno	85.0	85.0	90.0
Enugu	96.9	96.9	84.4
Gombe	97.0	93.9	87.9
Kaduna	97.4	100.0	97.4
Kano	100.0	96.7	76.7
Lagos	96.9	93.8	75.0
Ogun	100.0	90.3	77.4
Sokoto	96.9	93.8	96.9
Total	97.4	94.8	87.6
Childcare			
Akwa Ibom	100.0	100.0	93.8
FCT	96.3	92.6	100.0
Borno	80.0	80.0	90.0
Enugu	96.9	93.8	93.8
Gombe	81.8	78.8	90.9
Kaduna	92.1	94.7	97.4
Kano	70.0	66.7	93.3
Lagos	93.8	90.6	96.9
Ogun	87.1	83.9	96.8
Sokoto	100.0	93.8	96.9
Total	90.2	87.9	95.1
Adolescent health			
Akwa Ibom	96.9	96.9	100.0
FCT	77.8	85.2	92.6
Borno	75.0	80.0	80.0
Enugu	84.4	87.5	93.8
Gombe	81.8	72.7	78.8
Kaduna	71.1	76.3	94.7
Kano	30.0	30.0	66.7
Lagos	62.5	65.6	93.8
Ogun	90.3	87.1	90.3
Sokoto	90.6	93.8	100.0
Total	76.2	77.5	89.6

### Family planning

Prior to the COVID-19 pandemic and lockdown, 97.7% of the 307 PHCs offered family planning services. There was a slight decrease during the lockdown to 95.8%, and a further decrease after the lockdown to 92.5%. Within States, 90–100% of the sampled PHCs offered family planning services before the COVID-19 pandemic, and during the lockdown. After the lockdown, the percentage of facilities offering FP services slightly decreased in Akwa Ibom, FCT, Enugu, Gombe, Kaduna, Lagos, Ogun, and Sokoto.

### Antenatal care

This service includes all pre-natal services as recommended by the government of Nigeria [21]. Before the lockdown, 94.8% of all the sampled PHCs in the ten States offered antenatal care services. They all continued with this service during the lockdown, and after the lockdown, there was an increase to 97.7%. Within States, only Gombe experienced close to a 10% decrease during the lockdown.

### Delivery care

This refers to the management of labour and child birth [21]. The majority (81.8%) of the PHCs offered delivery care before the lockdown. The proportion increased slightly during the lockdown to 83.7% and 94.1% after the lockdown. Within States, all the sampled PHCs in the FCT offered delivery care before the lockdown, and between 74.2% and 94.7% of the PHCs in the various States offered delivery care except in Lagos where 37.5% reported offering delivery care. The percentage of PHCs offering intrapartum care remained stable or increased in all the States, during and after the lockdown except in Gombe where the proportion post-lockdown returned to the pre-COVID level.

### Postnatal care

Postnatal care includes all checks on a mother and child within 48 h after birth and 6 weeks after birth excluding immunization [21]. Postnatal care was offered in 88.9% of the PHCs, the percentage increased to 87% during the lockdown, and decreased to 82.7% after the lockdown. In the various States, between 73.3% and 100% of the facilities offered postnatal care. All the states except Borno, Kaduna, Lagos, and Ogun reported some decline during the lockdown. After the lockdown, the decrease continued in Enugu, Ogun, and Sokoto. Lagos reported a sharp decrease from 81.2% to 40.6%.

### Childhood immunization

This service includes all the routine immunization for children aged 0–23 months [21]. The majority (97.4%) of the PHCs offered childhood immunization before the pandemic. There was a slight decline to 94.8% during the lockdown and a nearly 10% decline after the lockdown. Except in Borno where 85% of the facilities offered childhood immunization, between 96.9 and 100% of the facilities in all the States offered this service before the pandemic. During the lockdown, Akwa Ibom, Gombe, Kano, Lagos, Ogun and Sokoto experienced a slight decrease. After the lockdown, Akwa Ibom remained at the lockdown percentage of 93.8%, whereas there was a decrease in the FCT, Enugu, Gombe, Kaduna, Kano, Lagos, and Ogun.

### Child care

This consists of all care for young children excluding immunization below age 10 [21]. Childcare was offered in 90.2% of the PHCs before COVID-19. There was a slight decrease to 87.9% during the lockdown and an increase to 95.1% after the lockdown. In the various States, all the sampled PHCs in FCT, Akwa Ibom and Sokoto offered childcare before the pandemic, and the least percentage was in Kano where 70% offered childcare. During the pandemic, all the States except Akwa Ibom, Borno, and Kaduna experienced some decrease in the proportion of PHCs that offered childcare. After lockdown, Akwa Ibom reported a slight decline, whereas other States experienced some increase.

### Adolescent health care

This includes all health services offered to people aged 10–18 years which include reproduction health services, nutrition, and dental care among others [21]. Adolescent health care services were offered in 76.2% of the 307 PHCs before the pandemic. During the lockdown, there was a slight increase to 77.5% and a 17.6% increase to 89.6% after the lockdown. Except in Kano where only 30% of the PHCs offered adolescent health service before the pandemic, between 62.5% and 96.9% offered adolescent health services in the other nine States. All the states reported some increase during the lockdown except Gombe, Kano, and Ogun, and after the lockdown, the increase continued in all the States.

### Mode of service delivery during the lockdown

Table 3 presents the distribution of the mode of service delivery during the lockdown. Although the sampled PHCs offered RMNCH services during the lockdown, 75.2% offered all the range of services (full-service), whereas 24.8% reported offering only selected services at specified time during the day (Partial). Within States, nearly half of the PHCs in Borno State offered partial services during the lockdown, and 20–34% operated partially in six States.

**Table 3:** Service delivery during the lockdown

State (No of PHCs)	Full service N (%)	Partial service N (%)
Akwa Ibom (32)	30 (93.8)	2 (6.2)
FCT (27)	23 (85.2)	4 (14.8)
Borno (20)	11 (55.0)	9 (45.0)
Enugu (32)	21 (65.6)	11 (34.4)
Gombe (33)	27 (81.8)	6 (18.2)
Kaduna (38)	27 (71.1)	11 (28.9)
Kano (30)	24 (80.0)	6 (20.0)
Lagos (32)	25 (78.1)	7 (21.9)
Ogun (31)	21 (67.7)	10 (32.3)
Sokoto (32)	22 (68.8)	10 (31.2)
Total (307)	231 (75.2)	76 (24.8)

### Service utilization

The number of clients who utilized the PHCs for family planning services decreased during the lockdown; after the lockdown, there was an increase of 3.2% from the pre-COVID-19 number. This pattern is similar for antenatal care, delivery care, postnatal care, childhood immunization, childcare, and adolescent health care. A similar pattern of decline during the pandemic lockdown was observed in most of the States, (See Additional file 1).

The total number of clients who utilized the various RMNCH services in the ten States (per week) before the pandemic, during, and after lockdown is presented in Table 4 with the mean and standard deviation. The test statistics from the Kruskal Wallis H test to determine statistically significant difference between States are also presented with the lowest and highest mean rank before the pandemic lockdown, during the lockdown and after the lockdown.

**Table 4:** Service Utilization – Number of clients per week

Service	Before COVID-19					During lockdown					After lockdown				
	No. of clients	Mean	Std. Dev	Test statistics χ2 df = 9	p-value	No. of clients	Mean	Std. Dev	Test statistics χ2 df = 9	p-value	No. of clients	Mean	Std. Dev	Test statistics χ2 df = 9	p-value
Family Planning	7384	24.1	32.4	55.98	< 0.001	3776	12.3	12.6	81.65	< 0.001	7620	24.8	31.8	67.09	< 0.001
Antenatal care	12744	41.5	71.0	94.20	< 0.001	6562	21.4	26.7	73.03	< 0.001	13177	42.9	83.1	98.25	< 0.001
Delivery Care	3307	10.8	15.0	69.94	< 0.001	2262	7.4	12.4	68.45	< 0.001	3991	13.0	23.4	87.15	< 0.001
Postnatal care	5925	19.3	36.0	60.79	< 0.001	3640	11.9	20.1	53.93	< 0.001	6013	19.6	35.3	71.94	< 0.001
Child Immunization	17024	55.5	73.2	44.45	< 0.001	10,064	32.8	53.1	25.32	0.003	17257	56.2	79.1	55.61	< 0.001
Child Care	8134	26.5	35.2	29.50	0.001	4782	15.6	23.5	41.50	< 0.001	8866	28.9	42.8	34.95	< 0.001
Adolescent Care	4952	16.1	23.7	48.33	< 0.001	4065	13.2	20.4	56.92	< 0.001	3921	12.8	22.8	51.22	< 0.001

Test statistics is from Kruskal Wallis test of the mean difference between States

The mean difference between States in the number of family planning clients was statistically significant before the pandemic, during lockdown, and after lockdown. There was a statistically significant difference between States in the number of antenatal care clients before the pandemic, during, and after the lockdown. The lowest mean rank was in Enugu in the three periods, and the highest was Kano before the pandemic, Gombe during lockdown, and Kano after lockdown.

The number of clients who utilized the PHCs in the ten States for delivery care differed significantly before the pandemic, during the lockdown, and after the lockdown. The number of clients who utilized the PHCs for postnatal care differed significantly by State before the pandemic, during the lockdown, and after the lockdown.

There was a statistically significant difference between the States in the number of clients who used the PHCs for childhood immunization, before the pandemic, during the lockdown, and after lockdown. Utilization of the PHCs for general childcare differed significantly by State before the pandemic, during the lockdown, and after lockdown. The number of clients who used the PHCs for adolescent care differed significantly by State before the pandemic, during the lockdown, and after the lockdown.

### Reported difficulties in service delivery

Many of the PHCs reported difficulties in service delivery during the pandemic lockdown (Table 5). Most challenges were reported in Akwa Ibom, Ogun and Kaduna, while Sokoto, Borno, and Lagos reported the least percentages of challenges. Stock-out of drugs was reported by 25.7% of the PHCs, stock-out of contraceptive products was reported by 25.1%, harassment by the law enforcement agents was reported by 76.9%, and transportation difficulties were reported in 55.8%. By contrast 26.1% reported no difficulties in service delivery during the period. Other reported difficulties in 63.8% of the PHCs included centre was shut for a month due to a COVID-19 patient detected, contact tracing of COVID-19 patients, difficulty controlling clients to abide by the COVID-19 prevention rules, harassment by hoodlums, high cost of transportation, inadequate supply of personal protective equipments (PPEs), no incentive from the government, fear of the risk of infection, limited clinic opening time due to curfew during the lockdown, no water, no toilet, low morale, and insults from patients who do not believe there is COVID-19.

**Table 5:** Percentage distribution of PHC facilities reporting selected difficulties during the lockdown by States

State	No difficulty	Stock out of drugs	Stock out of contraceptives	Harassment by law enforcement agents	Transportation difficulties		Others
Akwa Ibom	3.1	6.2	9.4	86.7	73.3		81.2
Borno	45.0	40.0	40.0	45.0	40.0		75.0
Enugu	18.8	25.0	18.8	37.5	56.2		62.5
Gombe	24.2	60.6	60.6	71.9	50.0		57.6
Kaduna	18.4	36.8	39.5	94.7	50.0		57.9
Kano	20.0	6.7	6.7	70.0	86.7		90.0
Lagos	40.6	6.2	6.2	80.6	45.2		59.4
Ogun	12.9	9.7	9.7	87.1	61.3		90.3
Sokoto	65.6	37.5	37.5	90.6	65.6		18.8
FCT	18.5	29.6	22.2	92.6	22.2		51.9
Total	26.1	25.7	25.1	76.9	55.8		63.8

### Availability of personal protective equipment (PPE)

Only 2% of the 307 PHCs reported the availability of gowns. Within States, the highest percentage was 15% in Borno, while six states had no gowns. Gloves were available only in 18% of the PHCs; and within States, the highest percentage was 33.3% in the FCT and the lowest was in Borno (5%). Most of the PHCs (90.1%) had hand sanitisers; the highest percentage was reported in Ogun State (96.8%) while the lowest was in Lagos (83.9%). A temperature checker was available in 94.1% of the facilities. Whereas 100% of the PHCs in Sokoto, Borno and Kaduna had temperature checker, only 80.6% had in Ogun State.

### Experience of cases of COVID-19 in the health facilities

Slightly above one in ten (10.6%) of the sampled PHCs identified clients with symptoms of COVID-19. The highest percentage was reported in Borno (40%), and the lowest was in Gombe with 0%.

## Discussion

The study was designed to investigate the experiences of PHCs in selected States of Nigeria on the management of RMNCH services and explore the potential effects of the pandemic in limiting the delivery and access to such services. We focused on PHCs given that they are the entry point and the first port of call into the Nigerian health care system, ensuring equitable and affordable access to services for all citizens [21, 22]. With respect to RMNCH services, we investigated the availability of the service, service utilization, challenges in service utilization, availability of PPEs for prevention of COVID-19, and case-reporting of COVID-19 in the health facilities.

The results showed an increased tendency for the PHCs to open for antenatal and delivery services, but less so for postnatal services. This is possibly due to the importance of pregnancy and delivery that had occurred before or during the pandemic which the health facilities identified as essential for continued service provision.

Most noteworthy was the slight decline in the number of health facilities offering family planning immunization services, and childcare during the period. Although insignificant, these declines have the potential to reduce the tenacity with which such services are offered in the PHCs, which could dampen the future effectiveness of family planning and immunization programs.

It was of interest that adolescent services did not decline during and after the lockdown period, and indeed, the offering increased in most of the States. This may be due to the importance attached to adolescent health services by the health facilities.

Evidence from studies in other settings indicate disruptions in sexual and reproductive health services occasioned by the pandemic. In a multi-method survey with respondents drawn from 29 countries, decreased access to contraceptives and abortion services due to a diversion of attention by the government and health facilities to COVID-19 was demonstrated. From the demand side, barriers during the lockdown such as lack of finances, fear of infection, lack of transport, and closure of clinics among others were reported [23]. A study in China reported evidence of disruptions in antenatal, delivery, and postnatal care, abortion services, and stock-out of contraceptives [24]. A study conducted in four sub-Saharan African countries reported significant increase in contraceptive need and use among women in a marital union during the early stage of the pandemic [25]. However, the study also indicated that the trend may change. Additionally, evidence from the perspectives of health providers indicate disruption in SRHR services due to the pandemic [26, 27]. In a study conducted in Ethiopia, Burkina Faso and Nigeria, slightly more that 30% disruption in child and maternal health services was reported by providers [28].

A further area we investigated was the extent to which services were utilized before and after the lockdown. This was obtained through reviewing weekly statistics on service utilization in the PHCs before, during, and after the lockdown. The results showed a 30–50% reduction in service utilization for family planning, antenatal, delivery, postnatal care, immunization, and childcare services during the lockdown as compared to the pre-pandemic period. Similar decline in service utilizations has been reported in other settings [29–31]. While service utilization for most components improved after the lockdown, adolescent health services continued to witness reduced counts in all the facilities after the lockdown. This may be due to the special nature of adolescents, their free mobility, and the fact that they have their own notions of health care utilization which may manifest as a result of the pandemic [32]. This aspect must be further investigated as adolescents are at high risk of physical, mental and social effects of the pandemic and gender-based violence which has been postulated to have an increased incidence as a result of the lockdown associated with COVID-19 [33, 34].

We investigated the challenges reported by the respondents as limiting their delivery of essential reproductive health services during the COVID-19 lockdown. Several anecdotal reports have featured challenges in health facilities as a major difficulty during the pandemic, but no substantive empirical evidence has yet been provided. Close to three-quarter of the health facilities in all States reported major challenges, with the majority reporting multiple challenges. Such challenges were mostly reported in Akwa Ibom, Gombe, Kaduna, Kano, Lagos and Ogun States. They ranged from “out of stock syndrome” (mostly in Gombe, Borno, and Sokoto), contraceptives not available (largely in Gombe, Borno, Kaduna, and Sokoto), and police harassment (in all States, especially in Kaduna, Sokoto and FCT, where more than 90% of the health facilities reported this outcome).

Other reported challenges with the delivery of services included difficulties with transportation and insufficient PPEs. With respect to PPEs, only 2% of the health facilities overall, and 16% reported the availability of protective gowns and hand gloves. By contrast, temperature checker and hand sanitisers were more frequently present.

If the health facilities are to be efficient in managing COVID-19, these challenges must be addressed on an ongoing basis. Our direct questioning on whether the PHCs had reported cases of the virus showed that up to 10% answered affirmatively, which means that the situation is real and requires urgent attention by managers of the facilities. Such measures should include the provision of guidelines for managing and triaging potential cases of the virus in the PHCs, the early referral of suspected cases to confirmatory, isolation and treatment sites, the provision of comprehensive PPEs and precautionary measures in the health facilities, staff motivation, and the training and re-training of PHC staff on COVID-19 management.

Efforts should be made to address the identified challenges by governments, non-governmental agencies, the private sector, and donor agencies working in low resource settings. Such efforts should include the development and adoption of policies and programs for the comprehensive provision of health care, including essential SRHR services during epidemics such as the COVID-19, and the strategic and continuous dissemination of information about the disease and its prevention.

### Study strengths and weaknesses

Although the curtailment of essential services due to the COVID-19 pandemic has been a major source of concern in Nigeria and other parts of Africa, to the best of our knowledge, this is one of the first empirical investigations of the nature and extent of this challenge. Our focus on PHCs in rural, semi-urban and urban settings ensures that the most basic unit of health care that is available to all citizens and where COVID-19 prevention measures can be universally delivered is one of the strong points of this study. Furthermore, our selection of 30 LGAs in 10 States, and 307 PHC health facilities for the study provides a good representation of all six geo-political zones of the country. This suggests that the results of the study can be generalized throughout the country.

However, on the downside, the study is limited by the fact that only one single key informant per health facility was interviewed. The interviews largely relied on recall of events such as challenges experienced in the health facilities which could not have been witnessed by only one informant. Recall bias was therefore a potential weakness of the study. The triangulation of these results with those obtained from focus group discussions with groups of health providers would have increased the accuracy of some of the results obtained. Nevertheless, the fact that the key informants supported the information they provided with existing records in the health facilities helped to improve the accuracy of the data obtained.

## Conclusion

The results of this study indicate that a large proportion of PHCs in Nigeria attempted to open for the provision of essential SRHR services during the COVID-19 pandemic lockdown. However, there was a significant reduction in clients’ utilization of services due to challenges experienced in service implementation such as stock-outs, and also to low demand for services by clients. Although PHC facilities reported cases of COVID-19, there was limited provision for PPE and other special offers that would motivate the health workers to optimize services for clients.

## Supplementary Information

**Additional file 1: Table S1.** Distribution Service Utilization by State – Number of clients per week.

## Data Availability

The data are available on request from the UNFPA country Office, Nigeria.

## Abbreviations

PTF: Presidential Task Force
SRHR: Sexual and reproductive health and rights
SRH: Sexual and reproductive health
PHC: Primary Health Centre
LGAs: Local Government Areas
FCT: Federal Capital Territory
NGOs: Non-governmental organizations
WHARC: Women’s Health and Action Research Centre
EVA: Education as a Vaccine
PPFN: Planned Parenthood Federation of Nigeria
IPs: Implementation partners
CSOs: Civil Society Organizations
ODK: Open Data Kit
RMNCH: Reproductive, maternal, new-born and child health
PPE: Personal protective equipment
