# Supplementary material for: Effect of COVID-19 pandemic on provision of sexual and reproductive health services in primary health facilities in Nigeria: a cross-sectional study
Source: Reprod Health. 2021 Aug 4;18:166. doi: 10.1186/s12978-021-01217-5 (PMC8334336; doi:10.1186/s12978-021-01217-5)
Supplement: Supplementary file 1 — Additional file 1: Table S1. Distribution Service Utilization by State – Number of clients per week. [file 12978_2021_1217_MOESM1_ESM.docx]

**FACILITY ASSESSMENT SURVEY**

**Service Utilization**

**Table S1. Distribution Service Utilization by State – Number of clients per week**

|  |  | **Family Planning** | | | | | **Antenatal care services** | | | | |
| --- | --- | --- | --- | --- | --- | --- | --- | --- | --- | --- | --- |
| **State** | **Period** | **Min.** | **Max.** | **Sum** | **Mean** | **Std. Dev.** | **Min.** | **Max.** | **Sum** | **Mean** | **Std. Dev.** |
| Akwa Ibom | Before Covid-19 | 3 | 100 | 707 | 22.1 | 22.5 | 1 | 200 | 1223 | 38.2 | 45.6 |
|  | During lockdown | 0 | 20 | 199 | 6.2 | 4.7 | 0 | 29 | 513 | 16.0 | 11.5 |
|  | After lockdown | 3 | 100 | 660 | 20.6 | 21.8 | 0 | 200 | 1042 | 32.6 | 46.1 |
|  |  |  |  |  |  |  |  |  |  |  |  |
| Borno | Before Covid-19 | 5 | 120 | 842 | 42.1 | 31.6 | 4 | 400 | 1656 | 82.8 | 87.0 |
|  | During lockdown | 4 | 40 | 310 | 15.5 | 9.2 | 1 | 250 | 745 | 37.3 | 57.6 |
|  | After lockdown | 5 | 130 | 1019 | 51.0 | 34.1 | 5 | 410 | 1633 | 81.7 | 85.0 |
|  |  |  |  |  |  |  |  |  |  |  |  |
| Enugu | Before Covid-19 | 0 | 50 | 281 | 8.8 | 9.7 | 0 | 70 | 463 | 14.5 | 14.8 |
|  | During lockdown | 0 | 20 | 108 | 3.4 | 3.9 | 0 | 50 | 265 | 8.3 | 11.5 |
|  | After lockdown | 0 | 50 | 275 | 8.6 | 10.2 | 0 | 70 | 494 | 15.4 | 15.9 |
|  |  |  |  |  |  |  |  |  |  |  |  |
| Gombe | Before Covid-19 | 2 | 100 | 810 | 24.6 | 19.3 | 4 | 160 | 2109 | 63.9 | 42.6 |
|  | During lockdown | 0 | 50 | 559 | 16.9 | 12.1 | 1 | 120 | 1457 | 44.2 | 30.9 |
|  | After lockdown | 3 | 80 | 873 | 26.5 | 19.2 | 5 | 200 | 2161 | 65.5 | 53.3 |
|  |  |  |  |  |  |  |  |  |  |  |  |
| Kaduna | Before Covid-19 | 4 | 75 | 782 | 20.6 | 16.2 | 0 | 80 | 794 | 20.9 | 14.8 |
|  | During lockdown | 2 | 50 | 482 | 12.7 | 10.0 | 0 | 42 | 537 | 14.1 | 9.3 |
|  | After lockdown | 5 | 100 | 720 | 19.0 | 18.6 | 3 | 80 | 730 | 19.2 | 13.8 |
|  |  |  |  |  |  |  |  |  |  |  |  |
| Kano | Before Covid-19 | 5 | 100 | 917 | 30.6 | 21.6 | 9 | 700 | 3686 | 122.9 | 173.3 |
|  | During lockdown | 0 | 24 | 278 | 9.3 | 6.7 | 2 | 140 | 928 | 30.9 | 29.5 |
|  | After lockdown | 4 | 150 | 1032 | 34.4 | 30.4 | 10 | 900 | 4207 | 140.2 | 212.9 |
|  |  |  |  |  |  |  |  |  |  |  |  |
| Lagos | Before Covid-19 | 2 | 450 | 1315 | 41.1 | 78.9 | 2 | 100 | 626 | 19.6 | 22.0 |
|  | During lockdown | 2 | 100 | 600 | 18.8 | 21.1 | 0 | 60 | 349 | 10.9 | 13.1 |
|  | After lockdown | 2 | 400 | 1314 | 41.1 | 70.3 | 0 | 68 | 621 | 19.4 | 17.8 |
|  |  |  |  |  |  |  |  |  |  |  |  |
| Ogun | Before Covid-19 | 2 | 105 | 500 | 16.1 | 19.6 | 0 | 65 | 578 | 18.7 | 19.5 |
|  | During lockdown | 0 | 25 | 244 | 7.9 | 7.6 | 0 | 40 | 305 | 9.8 | 10.7 |
|  | After lockdown | 3 | 70 | 485 | 15.7 | 15.2 | 0 | 60 | 545 | 17.6 | 18.2 |
|  |  |  |  |  |  |  |  |  |  |  |  |
| Sokoto | Before Covid-19 | 2 | 60 | 696 | 21.8 | 15.0 | 3 | 80 | 836 | 26.1 | 20.7 |
|  | During lockdown | 2 | 60 | 590 | 18.4 | 15.8 | 0 | 120 | 866 | 27.1 | 28.2 |
|  | After lockdown | 3 | 60 | 676 | 21.1 | 16.1 | 2 | 90 | 825 | 25.8 | 22.9 |
|  |  |  |  |  |  |  |  |  |  |  |  |
| FCT | Before Covid-19 | 5 | 88 | 534 | 19.8 | 18.8 | 5 | 100 | 773 | 28.6 | 24.8 |
|  | During lockdown | 2 | 45 | 406 | 15.0 | 13.3 | 0 | 72 | 597 | 22.1 | 21.7 |
|  | After lockdown | 3 | 105 | 566 | 21.0 | 20.8 | 3 | 134 | 919 | 34.0 | 31.4 |
|  |  |  |  |  |  |  |  |  |  |  |  |
| **Total** | **Before Covid-19** | **0** | **450** | **7384** | **24.1** | **32.4** | **0** | **700** | **12744** | **41.5** | **71.0** |
|  | **During lockdown** | **0** | **100** | **3776** | **12.3** | **12.6** | **0** | **250** | **6562** | **21.4** | **26.7** |
|  | **After lockdown** | **0** | **400** | **7620** | **24.8** | **31.8** | **0** | **900** | **13177** | **42.9** | **83.1** |

**Table 4 contd.**

|  |  | **Delivery care** | | | | | **Postnatal care** | | | | |
| --- | --- | --- | --- | --- | --- | --- | --- | --- | --- | --- | --- |
| **State** | **Period** | **Min.** | **Max.** | **Sum** | **Mean** | **Std. Dev.** | **Min.** | **Max.** | **Sum** | **Mean** | **Std. Dev.** |
| Akwa Ibom | Before Covid-19 | 0 | 20 | 241 | 7.5 | 6.3 | 0 | 200 | 1158 | 36.2 | 47.1 |
|  | During lockdown | 0 | 20 | 112 | 3.5 | 4.8 | 0 | 30 | 495 | 15.5 | 12.5 |
|  | After lockdown | 0 | 200 | 525 | 16.4 | 48.3 | 0 | 200 | 1032 | 32.3 | 46.6 |
|  |  |  |  |  |  |  |  |  |  |  |  |
| Borno | Before Covid-19 | 2 | 75 | 498 | 24.9 | 21.3 | 5 | 79 | 581 | 29.1 | 25.1 |
|  | During lockdown | 0 | 65 | 346 | 17.3 | 19.7 | 0 | 53 | 305 | 15.3 | 17.0 |
|  | After lockdown | 5 | 80 | 569 | 28.5 | 23.6 | 5 | 92 | 699 | 35.0 | 29.0 |
|  |  |  |  |  |  |  |  |  |  |  |  |
| Enugu | Before Covid-19 | 0 | 50 | 249 | 7.8 | 11.0 | 0 | 58 | 201 | 6.3 | 10.3 |
|  | During lockdown | 0 | 20 | 120 | 3.8 | 5.4 | 0 | 30 | 97 | 3.0 | 5.7 |
|  | After lockdown | 0 | 42 | 262 | 8.2 | 11.0 | 0 | 80 | 216 | 6.8 | 14.0 |
|  |  |  |  |  |  |  |  |  |  |  |  |
| Gombe | Before Covid-19 | 2 | 80 | 732 | 22.2 | 18.0 | 2 | 200 | 1184 | 35.9 | 40.2 |
|  | During lockdown | 1 | 100 | 686 | 20.8 | 22.9 | 1 | 150 | 939 | 28.5 | 32.2 |
|  | After lockdown | 2 | 120 | 992 | 30.1 | 26.8 | 2 | 130 | 1208 | 36.6 | 31.5 |
|  |  |  |  |  |  |  |  |  |  |  |  |
| Kaduna | Before Covid-19 | 0 | 35 | 209 | 5.5 | 6.9 | 0 | 73 | 591 | 15.6 | 16.5 |
|  | During lockdown | 0 | 13 | 123 | 3.2 | 3.5 | 0 | 80 | 381 | 10.0 | 14.0 |
|  | After lockdown | 0 | 22 | 192 | 5.1 | 4.7 | 2 | 70 | 506 | 13.3 | 14.2 |
|  |  |  |  |  |  |  |  |  |  |  |  |
| Kano | Before Covid-19 | 0 | 80 | 589 | 19.6 | 26.3 | 0 | 60 | 538 | 17.9 | 19.5 |
|  | During lockdown | 0 | 44 | 288 | 9.6 | 12.7 | 0 | 27 | 251 | 8.4 | 8.4 |
|  | After lockdown | 0 | 90 | 676 | 22.5 | 27.0 | 0 | 80 | 623 | 20.8 | 23.7 |
|  |  |  |  |  |  |  |  |  |  |  |  |
| Lagos | Before Covid-19 | 0 | 40 | 196 | 6.1 | 9.7 | 0 | 60 | 396 | 12.4 | 12.9 |
|  | During lockdown | 0 | 30 | 120 | 3.8 | 6.2 | 0 | 180 | 452 | 14.1 | 32.2 |
|  | After lockdown | 0 | 30 | 168 | 5.3 | 8.9 | 0 | 75 | 510 | 15.9 | 17.4 |
|  |  |  |  |  |  |  |  |  |  |  |  |
| Ogun | Before Covid-19 | 0 | 20 | 102 | 3.3 | 4.2 | 0 | 450 | 654 | 21.1 | 79.8 |
|  | During lockdown | 0 | 7 | 49 | 1.6 | 1.9 | 0 | 150 | 280 | 9.0 | 26.7 |
|  | After lockdown | 0 | 12 | 92 | 3.0 | 3.0 | 0 | 450 | 649 | 20.9 | 79.9 |
|  |  |  |  |  |  |  |  |  |  |  |  |
| Sokoto | Before Covid-19 | 0 | 25 | 240 | 7.5 | 6.6 | 0 | 37 | 341 | 10.7 | 7.0 |
|  | During lockdown | 0 | 20 | 198 | 6.2 | 5.9 | 0 | 29 | 288 | 9.0 | 6.7 |
|  | After lockdown | 0 | 20 | 223 | 7.0 | 6.4 | 0 | 37 | 329 | 10.3 | 7.6 |
|  |  |  |  |  |  |  |  |  |  |  |  |
| FCT | Before Covid-19 | 1 | 50 | 251 | 9.3 | 11.6 | 0 | 98 | 281 | 10.4 | 18.3 |
|  | During lockdown | 0 | 40 | 220 | 8.2 | 10.5 | 0 | 15 | 152 | 5.6 | 4.9 |
|  | After lockdown | 1 | 60 | 292 | 10.8 | 13.3 | 2 | 25 | 241 | 8.9 | 6.2 |
|  |  |  |  |  |  |  |  |  |  |  |  |
| **Total** | **Before Covid-19** | **0** | **80** | **3307** | **10.8** | **15.0** | **0** | **450** | **5925** | **19.3** | **36.0** |
|  | **During lockdown** | **0** | **100** | **2262** | **7.4** | **12.4** | **0** | **180** | **3640** | **11.9** | **20.1** |
|  | **After lockdown** | **0** | **200** | **3991** | **13.0** | **23.4** | **0** | **450** | **6013** | **19.6** | **35.3** |

**Table 4 contd.**

|  |  | **childhood immunization** | | | | | **Child care** | | | | |
| --- | --- | --- | --- | --- | --- | --- | --- | --- | --- | --- | --- |
| **State** | **Period** | **Min.** | **Max.** | **Sum** | **Mean** | **Std. Dev.** | **Min.** | **Max.** | **Sum** | **Mean** | **Std. Dev.** |
| Akwa Ibom | Before covid-19 | 8 | 300 | 1850 | 57.8 | 67.5 | 4 | 250 | 969 | 30.3 | 58.1 |
|  | During lockdown | 0 | 96 | 790 | 24.7 | 21.0 | 0 | 28 | 252 | 7.9 | 6.5 |
|  | After lockdown | 5 | 130 | 1280 | 40.0 | 27.9 | 1 | 300 | 928 | 29.0 | 71.4 |
|  |  |  |  |  |  |  |  |  |  |  |  |
| Borno | Before covid-19 | 5 | 300 | 1530 | 76.5 | 71.8 | 0 | 290 | 1265 | 63.3 | 70.4 |
|  | During lockdown | 0 | 180 | 570 | 28.5 | 40.2 | 0 | 150 | 418 | 20.9 | 33.8 |
|  | After lockdown | 7 | 300 | 1621 | 81.1 | 75.9 | 0 | 300 | 1728 | 86.4 | 96.2 |
|  |  |  |  |  |  |  |  |  |  |  |  |
| Enugu | Before covid-19 | 0 | 100 | 1007 | 31.5 | 22.6 | 1 | 60 | 581 | 18.2 | 17.0 |
|  | During lockdown | 0 | 50 | 537 | 16.8 | 13.4 | 0 | 30 | 291 | 9.1 | 8.5 |
|  | After lockdown | 0 | 100 | 1077 | 33.7 | 23.1 | 1 | 60 | 627 | 19.6 | 19.0 |
|  |  |  |  |  |  |  |  |  |  |  |  |
| Gombe | Before covid-19 | 15 | 150 | 2068 | 62.7 | 41.0 | 0 | 86 | 976 | 29.6 | 20.4 |
|  | During lockdown | 5 | 120 | 1479 | 44.8 | 31.0 | 0 | 52 | 657 | 19.9 | 14.7 |
|  | After lockdown | 15 | 157 | 2173 | 65.9 | 41.1 | 0 | 75 | 1078 | 32.7 | 20.8 |
|  |  |  |  |  |  |  |  |  |  |  |  |
| Kaduna | Before covid-19 | 2 | 112 | 1190 | 31.3 | 24.9 | 0 | 112 | 927 | 24.4 | 28.1 |
|  | During lockdown | 0 | 100 | 909 | 23.9 | 23.9 | 0 | 193 | 889 | 23.4 | 40.2 |
|  | After lockdown | 2 | 110 | 1224 | 32.2 | 24.9 | 0 | 110 | 978 | 25.7 | 28.2 |
|  |  |  |  |  |  |  |  |  |  |  |  |
| Kano | Before covid-19 | 5 | 500 | 2329 | 77.6 | 88.9 | 0 | 100 | 661 | 22.0 | 24.4 |
|  | During lockdown | 0 | 170 | 1028 | 34.3 | 36.9 | 0 | 120 | 378 | 12.6 | 22.2 |
|  | After lockdown | 5 | 600 | 2700 | 90.0 | 106.7 | 0 | 120 | 848 | 28.3 | 30.7 |
|  |  |  |  |  |  |  |  |  |  |  |  |
| Lagos | Before covid-19 | 2 | 800 | 2538 | 79.3 | 139.8 | 0 | 100 | 923 | 28.8 | 24.6 |
|  | During lockdown | 0 | 274 | 1325 | 41.4 | 54.6 | 0 | 60 | 473 | 14.8 | 16.0 |
|  | After lockdown | 3 | 800 | 2635 | 82.3 | 140.9 | 0 | 100 | 827 | 25.8 | 24.6 |
|  |  |  |  |  |  |  |  |  |  |  |  |
| Ogun | Before covid-19 | 8 | 270 | 1508 | 48.7 | 51.4 | 2 | 100 | 450 | 14.5 | 19.5 |
|  | During lockdown | 0 | 100 | 750 | 24.2 | 26.8 | 0 | 50 | 210 | 6.8 | 10.8 |
|  | After lockdown | 8 | 255 | 1377 | 44.4 | 49.5 | 0 | 100 | 447 | 14.4 | 19.5 |
|  |  |  |  |  |  |  |  |  |  |  |  |
| Sokoto | Before covid-19 | 0 | 90 | 891 | 27.8 | 17.5 | 5 | 180 | 906 | 28.3 | 34.2 |
|  | During lockdown | 0 | 150 | 873 | 27.3 | 28.8 | 5 | 160 | 850 | 26.6 | 33.1 |
|  | After lockdown | 0 | 90 | 811 | 25.3 | 19.2 | 3 | 158 | 851 | 26.6 | 29.9 |
|  |  |  |  |  |  |  |  |  |  |  |  |
| FCT | Before covid-19 | 8 | 383 | 2113 | 78.3 | 100.3 | 0 | 54 | 476 | 17.6 | 16.8 |
|  | During lockdown | 0 | 735 | 1803 | 66.8 | 142.3 | 0 | 52 | 364 | 13.5 | 13.0 |
|  | After lockdown | 8 | 708 | 2359 | 87.4 | 135.8 | 2 | 80 | 554 | 20.5 | 19.5 |
|  |  |  |  |  |  |  |  |  |  |  |  |
| **Total** | **Before covid-19** | **0** | **800** | **17024** | **55.5** | **73.2** | **0** | **290** | **8134** | **26.5** | **35.2** |
|  | **During lockdown** | **0** | **735** | **10064** | **32.8** | **53.1** | **0** | **193** | **4782** | **15.6** | **23.5** |
|  | **After lockdown** | **0** | **800** | **17257** | **56.2** | **79.1** | **0** | **300** | **8866** | **28.9** | **42.8** |

**Table 4 contd.**

| **Adolescent health care** | | | | | | |
| --- | --- | --- | --- | --- | --- | --- |
| **State** | **Period** | **Min.** | **Max.** | **Sum** | **Mean** | **Std. Dev.** |
| Akwa Ibom | Before covid-19 | 0 | 100 | 539 | 16.8 | 23.0 |
|  | During lockdown | 0 | 60 | 338 | 10.6 | 13.8 |
|  | After lockdown | 0 | 28 | 240 | 7.5 | 7.8 |
|  |  |  |  |  |  |  |
| Borno | Before covid-19 | 0 | 74 | 394 | 19.7 | 21.8 |
|  | During lockdown | 0 | 117 | 448 | 22.4 | 31.0 |
|  | After lockdown | 0 | 250 | 436 | 21.8 | 55.6 |
|  |  |  |  |  |  |  |
| Enugu | Before covid-19 | 0 | 30 | 263 | 8.2 | 6.8 |
|  | During lockdown | 0 | 39 | 187 | 5.8 | 8.1 |
|  | After lockdown | 0 | 20 | 187 | 5.8 | 5.2 |
|  |  |  |  |  |  |  |
| Gombe | Before covid-19 | 0 | 150 | 1098 | 33.3 | 33.1 |
|  | During lockdown | 0 | 135 | 991 | 30.0 | 30.4 |
|  | After lockdown | 0 | 85 | 864 | 26.2 | 21.9 |
|  |  |  |  |  |  |  |
| Kaduna | Before covid-19 | 0 | 110 | 500 | 13.2 | 18.8 |
|  | During lockdown | 0 | 110 | 367 | 9.7 | 18.0 |
|  | After lockdown | 0 | 154 | 528 | 13.9 | 27.8 |
|  |  |  |  |  |  |  |
| Kano | Before covid-19 | 0 | 50 | 211 | 7.0 | 14.2 |
|  | During lockdown | 0 | 50 | 124 | 4.1 | 10.0 |
|  | After lockdown | 0 | 50 | 133 | 4.4 | 10.3 |
|  |  |  |  |  |  |  |
| Lagos | Before covid-19 | 0 | 80 | 593 | 18.5 | 20.5 |
|  | During lockdown | 0 | 85 | 505 | 15.8 | 19.4 |
|  | After lockdown | 0 | 85 | 523 | 16.3 | 21.0 |
|  |  |  |  |  |  |  |
| Ogun | Before covid-19 | 0 | 80 | 363 | 11.7 | 14.7 |
|  | During lockdown | 0 | 30 | 239 | 7.7 | 7.8 |
|  | After lockdown | 0 | 80 | 274 | 8.8 | 15.3 |
|  |  |  |  |  |  |  |
| Sokoto | Before covid-19 | 0 | 80 | 599 | 18.7 | 18.0 |
|  | During lockdown | 0 | 70 | 609 | 19.0 | 16.8 |
|  | After lockdown | 0 | 100 | 555 | 17.3 | 20.1 |
|  |  |  |  |  |  |  |
| FCT | Before covid-19 | 0 | 220 | 392 | 14.5 | 41.6 |
|  | During lockdown | 0 | 130 | 257 | 9.5 | 24.7 |
|  | After lockdown | 0 | 46 | 181 | 6.7 | 10.2 |
|  |  |  |  |  |  |  |
| **Total** | **Before covid-19** | **0** | **220** | **4952** | **16.1** | **23.7** |
|  | **During lockdown** | **0** | **135** | **4065** | **13.2** | **20.4** |
|  | **After lockdown** | **0** | **250** | **3921** | **12.8** | **22.8** |
